# Aromatic clusters in protein–protein and protein–drug complexes

**DOI:** 10.1186/s13321-020-00437-4

**Published:** 2020-05-08

**Authors:** Esteban Lanzarotti, Lucas A. Defelipe, Marcelo A. Marti, Adrián G. Turjanski

**Affiliations:** 1 Departamento de Computación, Facultad de Ciencias Exactas y Naturales, Universidad de Buenos Aires, Buenos Aires, Argentina; 2 Departamento de Química Biológica, Facultad de Ciencias Exactas y Naturales, Universidad de Buenos Aires, Buenos Aires, Argentina; 3 IQUIBICEN/UBA-CONICET, Facultad de Ciencias Exactas y Naturales, Universidad de Buenos Aires, Buenos Aires, Argentina; 4 European Molecular Biology Laboratory Hamburg, Notkestraße 85, 22607 Hamburg, Germany

**Keywords:** Aromatic interactions, Protein–protein interactions, Protein–drug interactions

## Abstract

Aromatic rings are important residues for biological interactions and appear to a large extent as part of protein–drug and protein–protein interactions. They are relevant for both protein stability and molecular recognition processes due to their natural occurrence in aromatic aminoacids (Trp, Phe, Tyr and His) as well as in designed drugs since they are believed to contribute to optimizing both affinity and specificity of drug-like molecules. Despite the mentioned relevance, the impact of aromatic clusters on protein–protein and protein–drug complexes is still poorly characterized, especially in those that go beyond a dimer. In this work, we studied protein–drug and protein–protein complexes and systematically analyzed the presence and structure of their aromatic clusters. Our results show that aromatic clusters are highly prevalent in both protein–protein and protein–drug complexes, and suggest that protein–protein aromatic clusters have idealized interactions, probably because they were optimized by evolution, as compared to protein–drug clusters that were manually designed. Interestingly, the configuration, solvent accessibility and secondary structure of aromatic residues in protein–drug complexes shed light on the relation between these properties and compound affinity, allowing researchers to better design new molecules.

## Introduction

Aromatic rings are important residues for biological interactions and appear to a large extent as part of protein–drug and protein–protein interactions. π–π (both stacking and T-shape), anion-π and cation-π are the main interaction types described in the literature [1]. They are highly relevant for protein stability and molecular recognition processes due to their natural occurrence in phenylalanine, tyrosine, tryptophan and histidine residues. Aromatic rings are also often used in drug design since they contribute to optimizing both affinity and specificity [2] of drug-like molecules. Aromatic rings allow the generation of skeletons in lead compounds, that can be further optimized to achieve the target and off-target binding requirements [3, 4]. However, it is also important to note that higher aromatic ring count has been correlated with lower drug developability [5, 6]. Also, if a compound has poor solubility, reducing the number of aromatic rings is likely to be beneficial. This knowledge suggests that aromatic rings in drugs are resources that must be taken seriously.

Aromatic rings also appear in protein–protein interfaces playing a significant role as anchor residues. As shown by Rajamani et al. [7], binding interfaces usually present aromatic residues in the middle and, only a few of them, have aliphatic residues as anchors. Moreover, other studies [8, 9] showed that conservation of, mainly Trp and in lesser degree Phe and His, on the protein surface, possibly indicates a protein–protein interaction interface. In the last decades, there has been an increase in drugs designed to bind protein–protein interaction interfaces [10, 11]. These developments present additional difficulties compared to more traditional targets since it is not easy to find cavities that may be used as ligand-binding sites [12]. Designing peptides to inhibit protein–protein interactions is another promising strategy because it can be derived directly from protein sequences and have the capacity to cover larger areas compared with small molecules [13, 14]. Protein–protein interfaces usually have ‘hot spots’ that are smaller than the entire contact surface and have residues with high contribution to the free energy of binding [8, 15–17]. The identification of these ‘hot spots’ is of paramount importance as drugs are usually designed to bind them. Aromatic residues belong to this group of hot spots and are, therefore, relevant candidates for the design of protein–protein drug-like inhibitors [9].

The above-described relevance of aromatic interactions in protein structure, protein–protein, and protein–drug complexes, promoted the study of their structure and energetics. Previous work from our group, extended original studies of pairs of aromatic interactions, showing that inside proteins (intraprotein), aromatic rings (derived from Phe, Tyr, and Trp) are found forming clusters beyond aromatic dimers. These show an additive energetic nature and display particular structures [18, 19]. These clusters appear adopting the same motifs found for benzene clusters in gas phase, and when three or more aromatic residues form a cluster, usually, two of them are close in the protein in sequence bringing the other(s) from distant positions. Particularly relevant and exciting were the structures found for symmetric aromatic trimmers, which maximize the number of interactions, 3 for 3 residues [19]. However, the impact of aromatic clusters on protein–protein complexes is unknown. Particularly in those that go beyond a dimer, and even though several studies are focusing on protein–drug interactions [20, 21], a systematic study of aromatic interactions and their underlying cluster structures in protein–drug complexes has, to our knowledge, not been performed.

In this work, we studied two datasets of protein–drug and protein–protein complexes and systematically analyzed the presence and structure of their aromatic clusters. In particular, we compared those found in protein–drug complexes with those found in protein–protein interfaces. Additionally, we compare our results with the previously reported intraprotein dataset [19]. Our results show that aromatic clusters are highly present in protein–drug and protein–protein complexes, that the number of clusters and the average number of interactions is higher in the case of protein–protein compared to protein–drug and, that protein–drug interactions are enriched in face-to-face π-stacking conformations. Moreover, our analysis sheds light on the relation between aromatic cluster configuration and chemical characteristics with the compound K_i_. Finally, our results show that aromatic clusters in protein–protein could be more stable than protein–drug clusters as the former were optimized by evolution and not manually designed.

## Materials and methods

### Selection of protein–drug and protein–protein complexes

We constructed two datasets based on available structures in the PDB: (i) protein–drug complexes (protein–drug) and (ii) protein–protein complexes (protein–protein). First, we filtered the entire PDB in order to select only structures with ‘drug-like’ compounds. We removed those that appear in more than ten different crystals to eliminate natural compounds and also those having less than 100 Da to avoid small molecules that are too small. Additionally, we applied a 95% sequence identity clustering using CD-HIT [22] over the underlying set of protein targets.

We took a protein–drug complex for each cluster and each drug-like compound, to eliminate duplicate pairs when crystals have several structures and avoid biases due to a differential representation of protein families in the PDB. For the set of protein–protein complexes, we selected entries from the PDB that have only two chains and are explicitly mentioned in the PDB file header, which corresponds to a dimeric complex. Over these entries, we applied a clustering procedure at 95% of sequence identity ensuring not to repeat the same combination of two protein clusters (note: this procedure allows at least one homodimer for each sequence cluster).

For both datasets, only complexes with a resolution of 2.5 Å or below were kept for further analysis. Detailed statistics of the filtering can be seen in Additional file 1: Table S2.

### Aromatic clusters detection

First, we needed to detect aromatic rings. In protein molecules, benzene rings are found in Phe, Tyr, and Trp. Imidazole rings are found upon His and pyrrole in Trp. Aromatic ring selection in drug-like compounds was implemented using OpenBabel library [23] with python scripting (pybel) to get a set of atoms for every aromatic ring and to classify each ring by its chemical type (i.e., benzene, pyrrole, etc.). Each ring in a fused system is counted individually; for example, the indole group is defined as having two rings: benzene and pyrrole. We classified aromatic rings as residue rings (R) if they belong to a protein or as rings belonging to a drug compound (D). In this sense, aromatic interactions found in protein–protein interaction interfaces are made by two R rings from different protein subunits (chains) while protein–drug interactions are made between a D ring and an R ring.

**Fig. 1:**
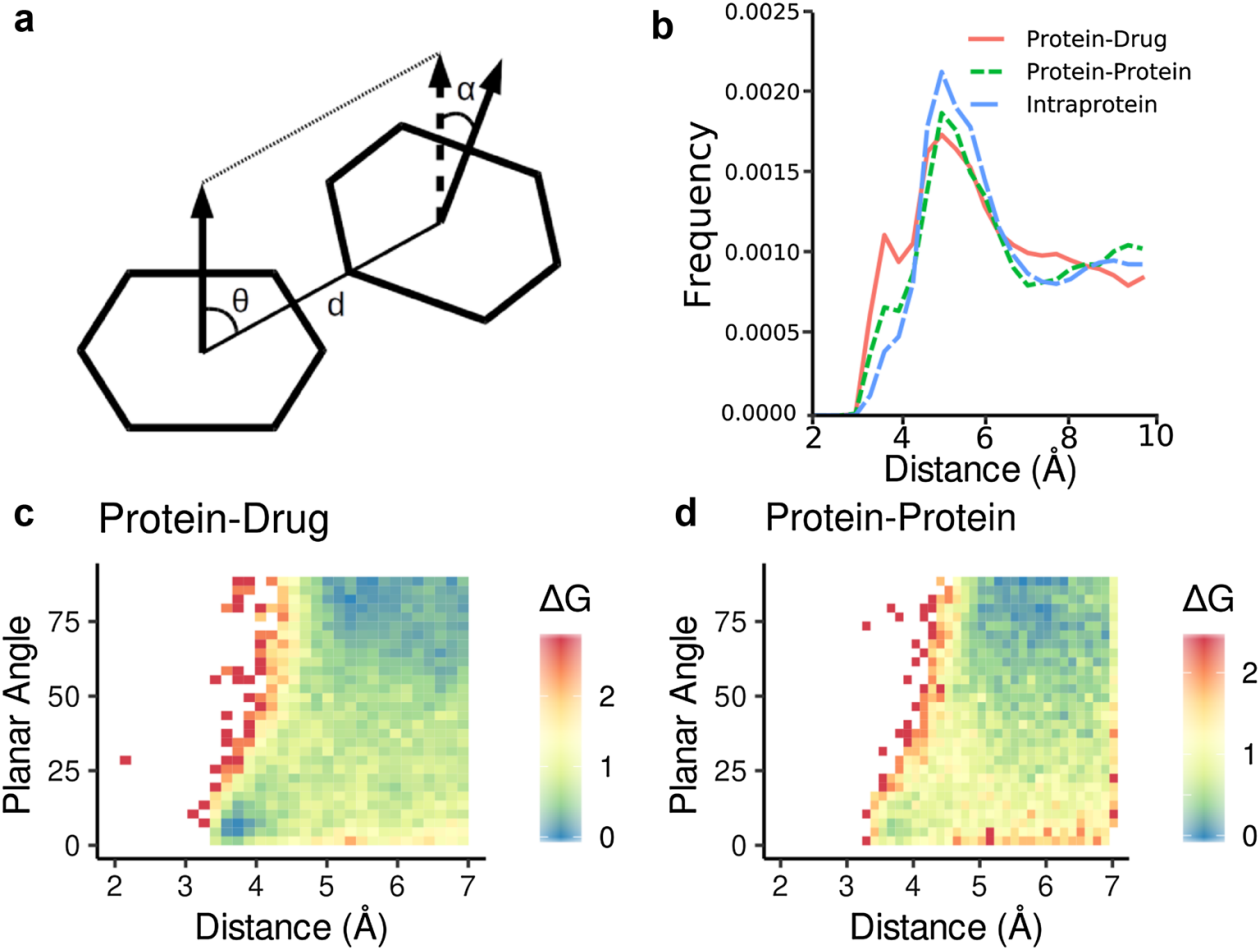
Aromatic interaction in protein–drug and protein–protein interactions. **a** Interaction scheme showing main aromatic interaction descriptors: distance (d), planar angle (α) and orientational angle (ө). **b** Radial distribution function for the distance between aromatic rings in protein–drug complexes (solid), protein–protein complexes (dashed) and intra-protein interactions (dotted). **c** Planar protein–drug interactions. **d** Planar protein–protein interactions. Color scale is a Delta G energy computed with Boltzmann Equation with a temperature of 298 K

Aromatic interactions are known to be mainly described by the distance between the centre of each ring [24]. Besides, angles can be used to describe aromatic interaction energetics better [25–27]. We detected aromatic interactions using the distance (d) between the geometric centre of each aromatic ring planes as defined in Fig. 1a, using a cutoff of 7.0 Å. Also, we used the planar angle (α) and the orientational angle (ө) to describe different π-π interactions such as π-stacking and T-shape, identifying in edge-to-edge, face-to-face and edge-to-face conformations.

In order to characterize three or more interacting rings, we defined the aromatic clusters as each connected component in the underlying graph of interactions. We analyzed every entry in the database by detecting aromatic clusters based only on drug–residue aromatic interactions and residue–residue interactions. Only these interactions have been included to count for the number of interactions reported and define each cluster. Then, the number of interactions of each aromatic cluster in protein–drug is calculated as the number of drug–residue interactions and the number of residue–residue interactions. In the case of protein–protein, only residue–residue interactions that correspond to two rings belonging to different chains were used for detection and, as well as in protein–drug, but residue–residue interactions between the same chain were included to count the total number of interactions.

Despite the filter of complexes that we performed after clustering, we wanted to evaluate if there were biases in protein families. We searched the PFAM database [28] and we found that both datasets cover a high diversity, as the most abundant in protein–drug were

1. Protein Kinase family (Pkinase:PF00069 and Pkinase_tyr:PF07714) 10.09%.
2. Trypsins (Trypsin:PF00089) 4.66%.
3. Nuclear Hormone Receptors (Hormone_recep:PF00104) 3.58%.
4. Retroviral Aspartyl Proteases (RVP:PF00077) 2.24%.

On the other hand, the protein–protein dataset the most abundant families were:

1. Immunoglobulins (V-set:PF07686 and C1-set:PF07654) 2.1%
2. Protein kinases (Pkinase:PF00069), 1.04%
3. Small GTPases Ras proteins (Ras:PF00071) 1.03%
4. Short-chain dehydrogenases (adh_short:PF00106) 0.91%

This means that no family is over-represented in neither protein–drug nor protein–protein.

Additionally, we have computed the amino acid composition of our two datasets and compared it with the whole PDB. No significant differences were found (Additional file 1: Figure S6).

### Structural properties of aromatic clusters

For each aromatic residue involved in a cluster, we computed the secondary structure using DSSP [29] and defined three major classes: Helix, Sheet, and Loop. Also, we calculated the preference of a given ring to be interacting with a residue in a particular secondary structure as the log (odds ratio) between the probability of this ring to be in that class and the class background probability. For example:

1 $$Preference\left( {sheet_{protein - protein} ,helix} \right) \, = log(\frac{ {P( helix|sheet_{protein - protein}})}{P(helix)})$$

where P(helix|sheet_protein–protein_) is the probability of a protein-protein residue in Sheet conformation to be interacting with a residue in Helix conformation. We also calculated the same preference for a given ring in a drug to be contacting a residue in a certain conformation.

Solvent Accessible Surface Area (SASA) was computed using the plug-in based on the Connolly surface algorithm as implemented in the VMD program [30]. The radius of the probe used was 1.4 Å. For each residue, we calculated the percentage of the exposed area as:

2 $$\% Exposed \, = \,\frac{eSASA}{iSASA}$$

with iSASA being the surface of the amino acid side chain in isolation and eSASA being the surface with the rest of the protein. Also, we calculated the percentage of the exposed surface that is in contact as:

3 $$\%\, in\, Contact = \frac{(eSASA-nSASA)}{eSASA}$$

with nSASA being the surface exposed in the context of the entire complex

### Affinity data mapping

Affinity data was taken from BindingDB [31] and mapped to the corresponding protein–drug complex. As of February 2020, BindingDB had 2291 protein–ligand crystal structures with binding affinity reported on that database where the identity of both the protein and the ligand matches perfectly.

## Results

### Occurrence and distribution of aromatic clusters in biomolecular complexes

To analyze the structure and prevalence of aromatic interactions in protein–drug (PD) and protein–protein (PP) complexes, we surveyed the PDB and the complexes were filtered to have only one for each pair of protein–protein and protein–drug complexes. We found and analyzed 10,231 protein–drug complexes and 4837 protein–protein complexes to identify the presence of protein–drug/protein–protein aromatic clusters. Aromatic clusters are defined by the presence of at least two (i.e. a dimer), aromatic rings that are interacting according to Materials and Method based on our previous work [19]. In the present case, we looked for clusters that have at least one ring on a different molecule. For detailed description and characterization of intra-protein aromatic clusters see our previous work [19]. The number of complexes with at least one aromatic cluster is 5908 in the case of protein–drug (57% of all protein–drug complexes). Amazingly, if we consider the complexes where the drug has an aromatic ring (ca. 66% of the drugs in the dataset fulfil this criterion), 87% of them are forming aromatic clusters. In the protein–protein complexes, we found 3048 with at least one aromatic cluster, which represents 63% of the dataset. The total number of clusters was 7236 (protein–drug) and 7717 (protein–protein), this means that protein–protein interaction sites have more than twice the number of clusters compared with drug binding sites, having 2.53 against 1.22 clusters per cluster. Accordingly, the total number of interactions found was 23,303 and 15,309 for protein–drug and protein–protein, respectively, which correspond to an average of 3.22 and 1.98 interactions per cluster for each case. (Table 1) Concerning cluster size, dimers are, as expected, the predominant type but trimers, tetramers and beyond are well represented.

**Table 1:** Aromatic–aromatic cluster complexes properties in protein–drug, protein–protein and intraprotein

Property	Protein–drug	Protein–protein	Intraprotein
≥ 1 Aromatic cluster complexes	5908	3048	9760
Number of clusters	7236	7717	73,312
Average number of clusters	1.22	2.53	7.51
Number of interactions	23,303	15,309	277,797
Average number of Interactions per cluster	3.22	1.98	3.78
Proportion of interacting aromatic residues (%PHE, %TYR, %TRP, %HIS)	34.8%, 26.5%, 21.7%, 16.8%	36.7%, 25.3%, 20.7%, 17.1%	35.2%, 24.5%, 25.4%, 14.6%
Proportion of secondary structures for interacting residues (%Loop, %Sheet, %Helix)	43.4%, 23.6%, 32.9%	34.3%, 22.4%, 43.2%	32.5%, 27.3%, 40.1%
Average % exposure of aromatic residues	17.29	34.63	9.18

Interestingly, dimers are over-represented in protein–protein (59%) compared to protein–drug (31%) complexes (Additional file 1: Figure S1A). As expected, the number of interactions increases with the cluster size quite evenly for both datasets for small cluster sizes. Nevertheless, for big clusters (≥ 6), the number of interactions is higher for the protein–drug group than the protein–protein group (Additional file 1: Figure S1B).

For the sake of comparison, we also look for intraprotein clusters in the protein–drug dataset, and found 9760 clusters (shown in Table 1), which represents 95% of the dataset. As expected, the number of clusters per complex is higher than in the other two cases (protein–drug and protein–protein) because of the volume analyzed. Protein cores are bigger than protein–protein interfaces and drug binding sites. However, the average number of interactions in intraprotein clusters is comparable to that of protein–drug complex clusters, and both cases are around double that in the protein–protein clusters.

As aromatic trimmers adopt two different conformations in space, Symmetric (Sym) and Ladder (Lad) we analyzed how they behave in Protein–drug and Protein–Protein interactions (see Additional file 1: Figure S4). In particular, most of the protein–drug timers (78%) are in the Ladder structure and the same is for protein–protein Ladder trimers (79%). In both datasets, the number trimers seem to be equally balanced in terms of structural choice. (Additional file 1: Table S1).

### Aromatic residues interact slightly different in protein–drug vs protein–protein complexes

To characterize the structure of each cluster we determined two key parameters (Fig. 1a): The distance between the centre of each aromatic ring and the planar angle (α), which is the angle formed between the planes of the two rings. We compared aromatic interactions in protein–drug and protein–protein complexes, as well as those found inside proteins, in terms of both parameters. In all cases, the plot is very similar (Fig. 1b), with a broad wide peak at ca. 5 Å, which is the optimal average distance between two aromatic rings, in a T-shape like orientation (optimal angle is ca. 75°, see Fig. 1c and d). Surprisingly, all distributions also show a minor narrow peak at ca. 3.75 Å, but the relative intensity of it is different in all three cases. It is small in intra-protein clusters, slightly larger in protein–protein and largest in protein–drug complexes. This peak corresponds to the distance of optimum π-stacking interaction according to McGaughey [26], and therefore our data suggest that stacking interactions are favoured in protein–drug complexes. This is confirmed by looking at the planar angle vs. distance plot for protein–drug and protein–protein (Fig. 1c and d) showing that π-stacking conformations, a planar angle close to zero degrees, are enriched in protein–drug compared to protein–protein complex.

**Fig. 2:**
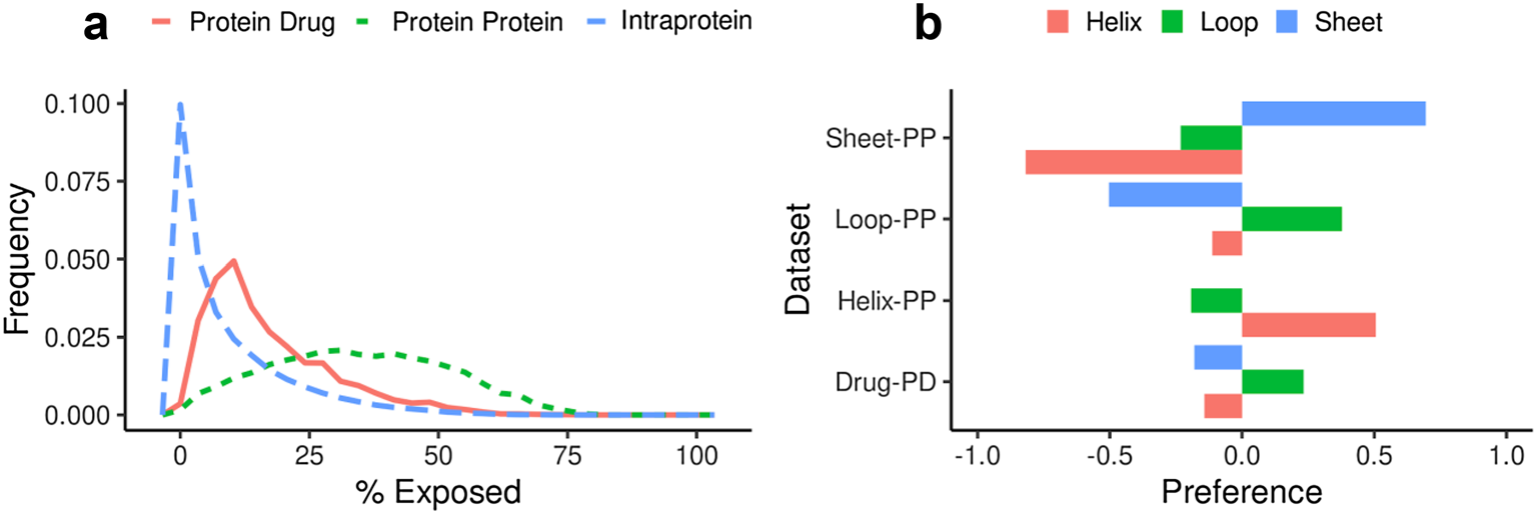
Structural properties of aromatic interacting residue and their secondary structure preference. **a** Percentage of exposed surface distribution for residues in protein–drug clusters (solid), protein–protein clusters (dashed) and intra protein clusters (dotted) **b** Secondary structure preference. On top, the three preferences for interacting residues in protein–drug, and below, the preferences for residues in protein–protein

In Fig. 2a, we compared the solvent exposure (or SASA) percentage for the rings containing residues in each group. As expected, aromatic residues inside the protein core are barely exposed. While residues in protein–drug are more exposed than protein core residues and the exposure of protein–protein residues is more significant and shows a wider range of solvent-accessible surface. In Additional file 1: Figures S2A and S2B, we compare the relationship between the solvent-accessible surface and the contact surface to understand how much surface is devoted to the aromatic interaction. Although we observed a solvent accessible surface spanning a wide range of values, the contact surface is mainly distributed at high values with the maximum of the distributions at 100% in both sets which means that the aromatic residues use all the available surface to establish the clusters. Figure 2b shows how the interacting rings of the clusters are distributed in the different secondary structure elements. This panel shows that aromatic residues interacting with drugs have a preference to be in loops and less likely to be in a structured region. The same is true for residues forming protein–protein complexes, residues in loops have a strong preference to be interacting with residues in loops from the partner protein.

Moreover, in protein–protein, secondary structure matches between interaction partners. Residues in sheet or helix have a preference to contact residues in the same secondary structure (sheet with sheet and helix with helix). These residues also present an inverse preference to be in contact with residues in the other secondary structures. Although the preference is slightly higher for sheet conformation, as aromatic trimmers have been reported to stabilize the structure of beta-sheets [32].

### Binding affinity dependence on aromatic interactions

**Fig. 3:**
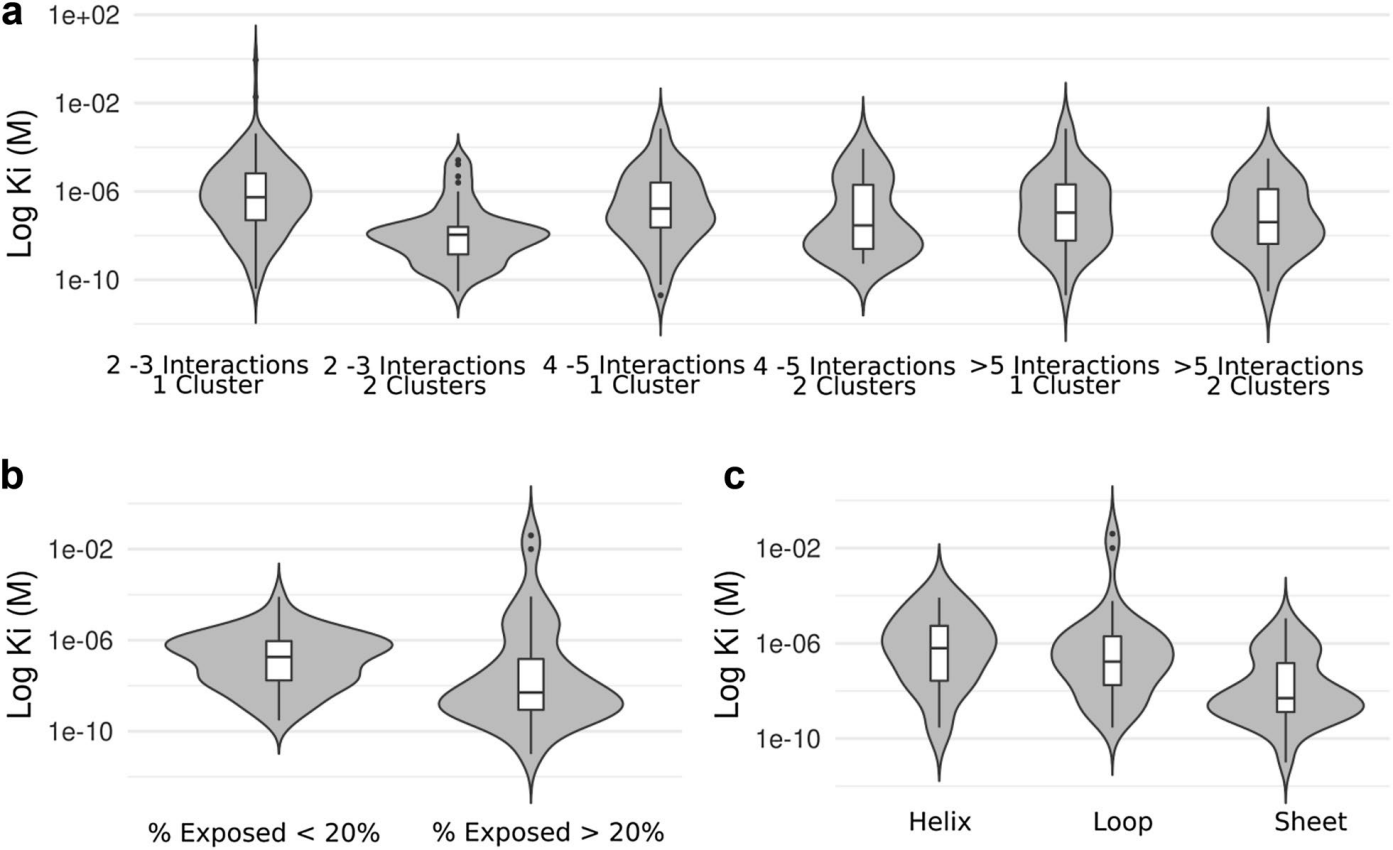
Aromatic clusters and binding affinity. **a** From left to right, violin plots are displayed increasing the number of interactions alternating (2 or 3, 4 or 5, 5 or more interactions) between drugs having 1 cluster and drugs with 2 clusters. Ki is expressed in molar units and y-axis is displayed in logarithmic scale. **b** Violin plot of Binding affinity of drugs interacting with a residue having less and more than 20% of the exposed surface. **c** Violin plot of drug binding affinity for drugs interacting with aromatic residues in different secondary structures

We now turn our attention to the relationship of different aromatic cluster properties and ligand affinity in the protein–drug complexes. We looked for binding affinity information using BindingDB [31] assigning (when possible) the K_i_ associated with a given complex in the protein–drug dataset. This assignment resulted in 358 protein–drug complexes with annotated binding affinity which have aromatic clusters with more than 2 ring interactions and with a resolution higher than 2.5 Å. First, in Fig. 3a, we display violin plots showing how K_i_ distributions vary as a function of the number of clusters (1 or 2) and the total number of ring interactions. First, as expected, when we look at complexes having only one cluster, the average K_i_ slightly decreases as the number of interactions increases. The trend is, however, not found when two clusters are present. Thus, no improvement in affinity is observed by adding more interactions. Also, comparing complexes with two or three interactions, if these are established using two clusters (Fig. 3a) the average K_i_ is improved about ten times to those cases having one cluster (Fig. 3a). In other words, it is better to anchor the drug through more than one cluster (or interaction site). However, this is only possible when the drug displays more than one aromatic ring.

As shown in Fig. 3b, we found that residues that are more than 20% exposed, interact with drugs having significantly lower binding affinity than those exposed less than 20% (Fig. 3b). Example conformations can be seen in Additional file 1: Figure S3A and S3B.

Now, we can ask if these properties have some relation with binding affinity to drugs.

Interestingly, as shown in Fig. 3c, we found that the secondary structure presents great differences. Residues in helix conformation (Additional file 1: Figure S3D) have weaker binding affinity than residues in sheets (Additional file 1: Figure S3C) and loops (Additional file 1: Figure S3E), and surprisingly residues in sheets have significantly better binding affinity than the other two cases. This difference contrasts heavily with the fact mentioned before that drugs have a negative preference for contacting residues in sheet conformation.

## Discussion

In this work, we studied aromatic clusters over two sets showing relevant biomolecular interactions, protein–drug, and protein–protein complexes. We found that in protein–drug complexes, around 74% of aromatic containing drugs form at least one aromatic interaction with its target. The number of clusters and the average number of interactions is higher in the case of protein–protein compared to protein–drug, which can be explained since protein–protein interactions have been optimized through evolution meanwhile interactions with drugs are designed artificially. Also, looking at angle distributions deduced from aromatic interactions, we found that aromatic rings in protein–drug interact reaching lower-energy conformations than in protein–protein, which are very similar to those found in protein cores [19]. Meanwhile, protein–drug interactions are enriched in face-to-face π-stacking conformations compared to protein–protein probably because the former have restrictions imposed by the protein backbone (Fig. 1). Because aromatic rings in drugs interact with its protein target with more degrees of freedom, allowing aromatic rings to interact more tightly.

According to solvent accessibility and secondary structure, residues in protein–drug and protein–protein are different. Regarding their secondary structure, rings in drugs are found to interact mainly with residues in loops discouraging other types of secondary structures (Fig. 2). However, residues in protein–protein interfaces match the same secondary structure. Additionally, clusters in protein–protein interfaces are formed by residues having more exposed surfaces than those residues found binding drugs. Also, because the contact surface is one of the main parameters that describe hydrophobic interactions (due to the hydrophobic effect, with more surface buried, there are more water molecules excluded). This difference in exposure could explain why aromatic clusters in protein–protein could be more stable than protein–drug clusters.

Analyzing affinity, we found that clusters with two interactions bind with lower K_i_ values when they are established by two separated clusters rather than only one. For the case with two clusters, the affinity is between micromolar (μM) and nanomolar (nM) (Fig. 3). When four or five interactions are achieved, the addition of more interactions does not change binding significantly. When looking at aromatic rings individually by plotting the number of rings in a compound vs the interaction affinity (Ki) (Additional file 1: Figure S5), we see a similar tendency to a plateau around four to five rings. Nevertheless, the appearance of bimodality on > 5 rings argues that the addition of more rings could increase affinity in a system-dependant manner.

It was previously argued that having too many aromatic rings decreases drug developability [4, 5]. Our results on fused systems energetics support the idea that fused rings should not be the first choice to increase compound affinity. It is known that drugs with more than one aromatic system are rarely produced [33]. In this sense, the location of aromatic systems in a drug should be optimized according to the outstanding interactions with its target when more than one aromatic residue is available. Keeping the number of aromatic rings low but increasing the number of interactions by increasing the number of clusters and trying to avoid fused systems.

## Conclusions

In the present work, we have analyzed the occurrence of aromatic interactions in the structures deposited in the Protein Data Bank, focusing mostly on higher-order interactions that go beyond the pure dimer. We found that there are some differences in preference of interaction between protein–drug and protein–protein, while the former can adopt a traditional Pi-stacking interaction as well as T-shaped. The latter mostly prefers traditional T-shaped. On the affinity side, it is better to have more clusters with the same number of interactions. This information is valuable for optimization campaigns where medicinal chemists have to make decisions on which modifications to synthesize and test.

## Supplementary information

**Additional file 1.** Supplementary tables and figures.

**Additional file 2.** Protein-protein interactions with PDBID, chain and UniProtID.

**Additional file 3.** Protein-drug interactions with PDBID, chain, residue name and Ki data if available.

## Data Availability

The  SQL database and the scripts to compute the statistics are available at https://github.com/elanzarotti/aromatics. For simple access to the data Additional files 2 and 3 are provided with detected protein-protein and protein-drug aromatic interactions.
