# Supplementary material for: Aromatic clusters in protein–protein and protein–drug complexes
Source: J Cheminform. 2020 May 8;12:30. doi: 10.1186/s13321-020-00437-4 (PMC7206889; doi:10.1186/s13321-020-00437-4)
Supplement: Supplementary file 1 — Additional file 1. Supplementary tables and figures. [file 13321_2020_437_MOESM1_ESM.pdf]

Supplementary information for

Aromatic clusters in protein-protein and protein-drug complexes.

Esteban Lanzarotti[1,#], Lucas A. Defelipe[2,3,4,#], Marcelo A. Marti[2,3], Adrián G. Turjanski[2,3*]

[1] Departamento de Computación, Facultad de Ciencias Exactas y Naturales, Universidad de Buenos Aires, Buenos Aires, Argentina.
[2] Departamento de Química Biológica, Facultad de Ciencias Exactas y Naturales, Universidad de Buenos Aires, Buenos Aires, Argentina.
[3] IQUIBICEN/UBA-CONICET, Facultad de Ciencias Exactas y Naturales, Universidad de Buenos Aires. Buenos Aires, Argentina.
[4] European Molecular Biology Laboratory Hamburg, Notkestrasse 85, D-22607, Hamburg, Germany.

* To whom correspondence should be addressed: adrian@qb.fcen.uba.ar
#Both authors contributed equally

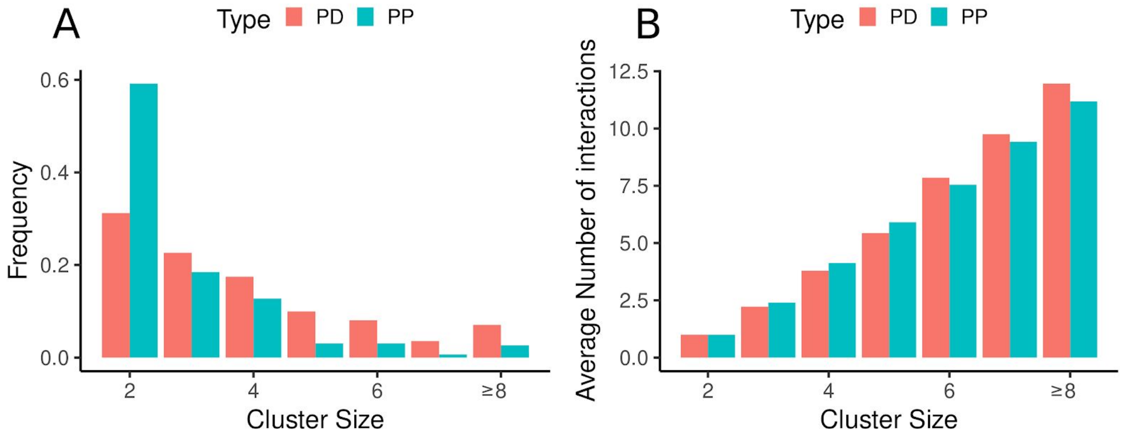

Figure S1. Aromatic clusters size and average interactions A) Aromatic cluster frequency for each dataset, Red for Protein-Drug, cyan for Protein-Protein. B)The average number of interactions by cluster size, Red for Protein-Drug, cyan for Protein-Protein.

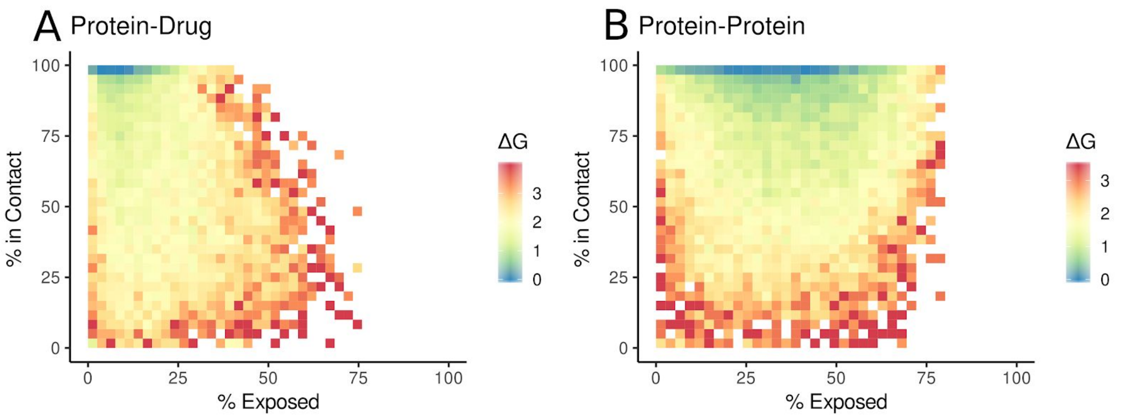

Figure S2. Solvent Accessibility and Contact Surface. A) % in Contact vs % Solvent-exposed for aromatic residues interacting with drugs. B) % in Contact vs % Solvent-exposed for aromatic residues interacting in protein-protein interfaces. The color scale is a Delta G energy computed with Boltzmann Equation with a temperature of 298K.

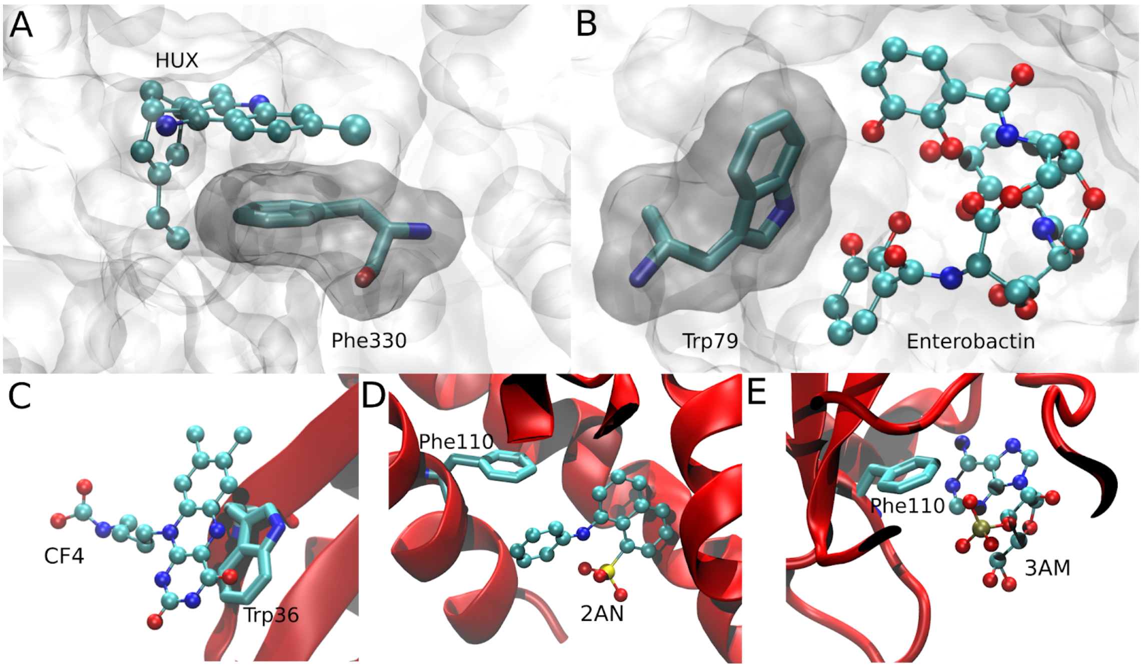

Figure S3. Structural examples from Figure 3. A) Example of a < 20% exposed aromatic residue. PDBID 1E66 F) Example of a > 20% exposed aromatic residue. 3CMP G) Aromatic residue in Sheet conformation. 2VKG H) Aromatic residue in Helix conformation. 1OW4 I) Aromatic residue in Loop conformation. 3AGN

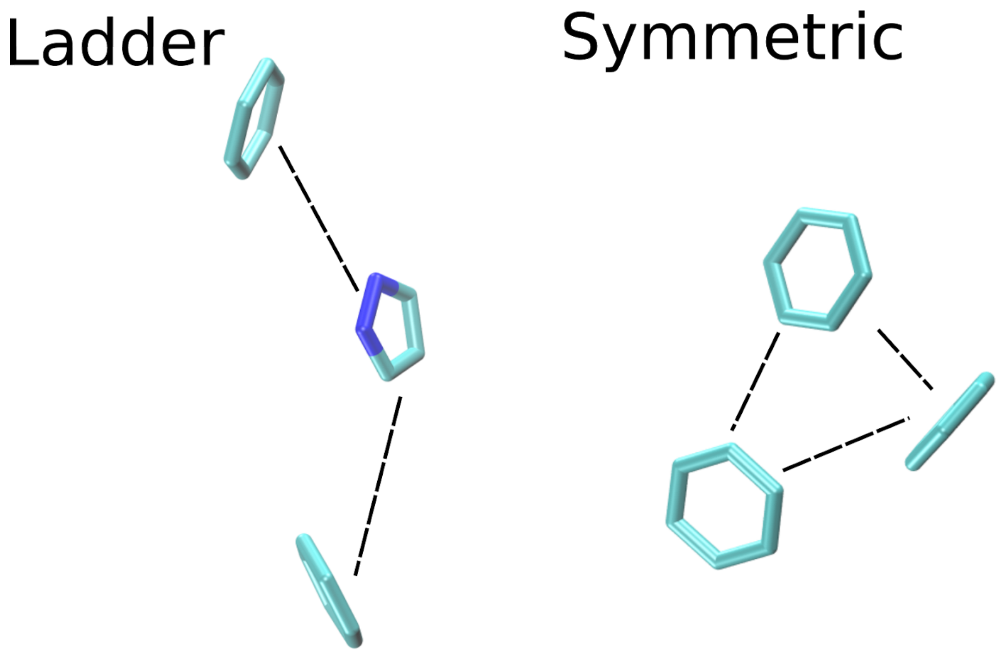

Figure S4. Conformations of aromatic trimers in protein-drug interactions. **Left.** Ladder structure of Phe 80, the Pyrazole ring of AT7519 and Phe 82 of *Hs*CDK2 complex (PDBId 2VTQ). **Right.** Symmetric structure of Phe 24, Phe 146 and Benzene ring of Penicillin G of *Ec.* Penicillin Acylase (PDBId 1FXV). Only aromatic rings are shown. The dashed line indicates interactions between the aromatic rings.

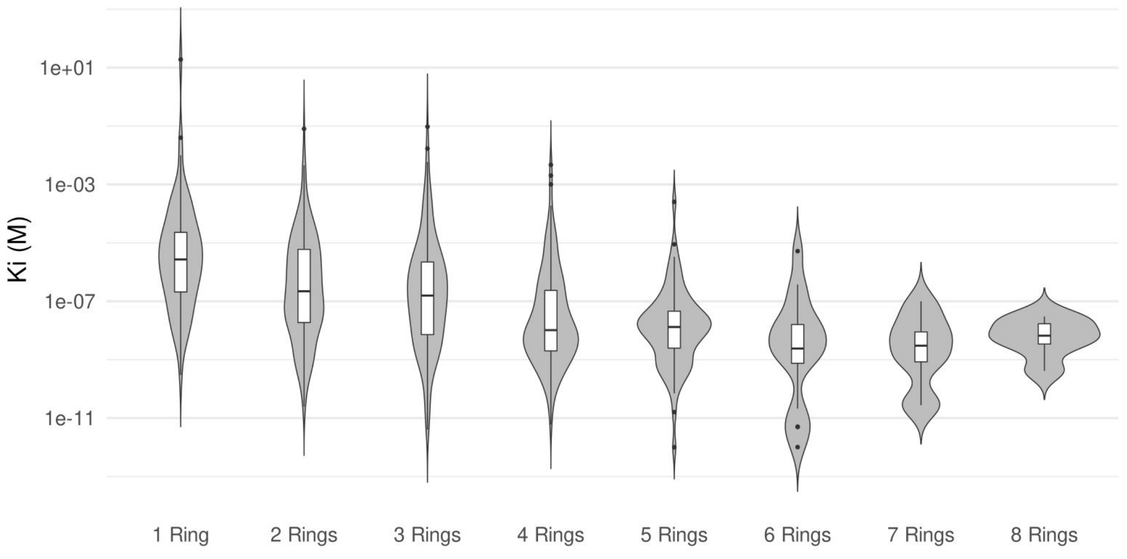

Figure S5. Number of aromatic rings in a protein-drug complex against interaction affinity (Ki). Ki is plotted in logarithmic scale.

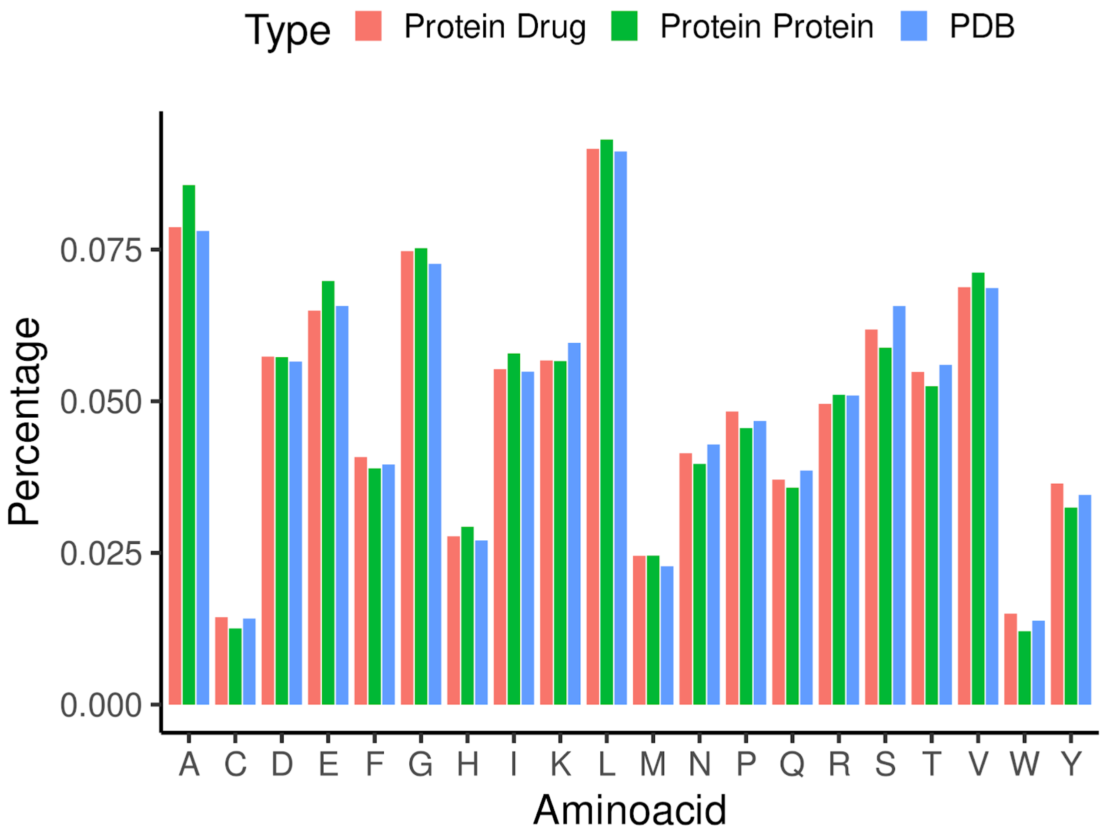

Figure S6. Database aminoacid composition bias. Average aminoacid composition of the proteins in the Protein-Drug and Protein-Protein datasets compared to the whole Protein Data Bank (PDB).

Type of trimer	Protein-Drug	Protein-Protein
LAD Trimers	1,283	1,126
SYM Trimers	358	294

Table S1. Number of Aromatic trimers classified by structural properties.

**Protein-drug (PD)**	
**PDBs with ligands appearing in more than 10 different crystals.**	**20610**
**Removing ligands having less than 100 Da.**	**19422**
**Redundancy elimination applying a 95% sequence identity.**	**13302**
**Only high-quality crystals with less than 2.5 resolution.**	**10231**
**Protein-protein (PD)**	
**PDBs having only two chains**	**23330**
**Explicitly mentioned as DIMER in REMARK 350 header**	**11097**
**Redundancy elimination applying a 95% sequence identity.**	**6224**
**Only high-quality crystals with less than 2.5 resolution.**	**4837**

Table S2. Filtration steps used to produce protein-drug and Protein-protein datasets.

Aromatic ring name	Number in the PD dataset
benzene	11404
pyrimidine	2372
pyridine	1653
imidazole	1260
pyrrole	1004
NOT SPECIFIED	579
thiophene	366
pyrazole	293
thiazole	253
pyrazine	232
Pyrazole anion	227
furan	179
isoxazole	116
oxazole	78
pyridazine	63
1,2,4-triazole	56
Thiazole cation	47
1,3,4-triazole	42
1,2,3-triazole	38
imidazole_cation	37
1,2,4-oxadiazole	29
1,3,4-thiadiazol	28
1,2,3,5-tetrazole	28
1,2,4-triazole_a	17
1,2,3-triazole_a	12
1,2,3,4-tetrazole	10
1,3,4-triazole_c	6
isothiazole	6
Oxazole cation	5
1,2,3-thiadiazol	4
Isoxazole cation	2
Isothiazole cation	1

Table S3. Number of rings by type in the protein-drug dataset.
